# Shared and distinct factors underlying in-hospital mobility of older adults in Israel and Denmark (97/100)

**DOI:** 10.1186/s12877-022-03636-w

**Published:** 2023-02-03

**Authors:** Anna Zisberg, Efrat Shadmi, Ove Andersen, Ksenya Shulyaev, Janne Petersen, Maayan Agmon, Efrat Gil, Nurit Gur-Yaish, Mette Merete Pedersen

**Affiliations:** 1 The Cheryl Spencer Department of Nursing, Faculty of Social Welfare and Health Sciences, University of Haifa, Mount Carmel, Haifa, 31905 Israel; 2 Center of Research & Study of Aging, University of Haifa, Haifa, Israel; 3 The Emergency Department, Copenhagen University Hospital Amager and Hvidovre, Hvidovre, Denmark; 4 Department of Clinical Research, Copenhagen University Hospital Amager and Hvidovre, Hvidovre, Denmark; 5 Department of Clinical Medicine, University of Copenhagen, Copenhagen, Denmark; 6 The Minerva Center On Intersectionality in Aging (MCIA), Faculty of Social Welfare and Health Studies University of Haifa, Haifa, Israel; 7 Center for Clinical Research and Prevention, Copenhagen University Hospital Bispebjerg and Frederiksberg, Frederiksberg, Denmark; 8 Geriatric Unit, Clalit Health Services, Haifa and West Galilee, Faculty of Medicine, Technion, Haifa, Israel; 9 Oranim Academic College of Education, Kiryat Tivon, Israel; 10 Physical Medicine and Rehabilitation Research-Copenhagen, Copenhagen University Hospital, Hvidovre, Hvidovre, Danmark

**Keywords:** Accelerometry, Risk factors, Function

## Abstract

**Background:**

Low in-hospital mobility is widely acknowledged as a major risk factor in acquiring hospital-associated disabilities. Various predictors of in-hospital low mobility have been suggested, among them older age, disabling admission diagnosis, poor cognitive and physical functioning, and pre-hospitalization mobility. However, the universalism of the phenomena is not well studied, as similar risk factors to low in-hospital mobility have not been tested.

**Methods:**

The study was a secondary analysis of data on in-hospital mobility that investigated the relationship between in-hospital mobility and a set of similar risk factors in independently mobile prior to hospitalization older adults, hospitalized in acute care settings in Israel (*N* = 206) and Denmark (*N* = 113). In Israel, mobility was measured via ActiGraph GT9X and in Denmark by ActivPal3 for up to seven hospital days.

**Results:**

Parallel multivariate analyses revealed that a higher level of community mobility prior to hospitalization and higher mobility ability status on admission were common predictors of a higher number of in-hospital steps, whereas the longer length of hospital stay was significantly correlated with a lower number of steps in both samples. The risk of malnutrition on admission was associated with a lower number of steps, but only in the Israeli sample.

**Conclusions:**

Despite different assessment methods, older adults’ low in-hospital mobility has similar risk factors in Israel and Denmark. Pre-hospitalization and admission mobility ability are robust and constant risk factors across the two studies. This information can encourage the development of both international standard risk evaluations and tailored country-based approaches.

## Introduction

Low in-hospital mobility has been identified as one of the strongest modifiable predictors of hospital associated functional decline [1–4], cognitive decline [5, 6], and even two-year mortality [7]. Studies have reported that low mobility during hospitalization is associated with immediate and long-term negative outcomes, not only in frail older adults, but also in independently functioning older patients [2, 3]. Research targeting a broad range of countries, patient populations, and healthcare systems consistently reports very low levels of in-hospital mobility among older patients [2, 8–11]. One study found the number of steps taken per day doubled immediately after discharge, suggesting older adults underuse their physical capacity during hospitalization [12].

Studies report a varied set of potential predictors of low in-hospital mobility, from patient characteristics to hospital and departmental policies and practices [11, 13–21]. Relevant departmental policies and practices include nursing practice and culture [22] and specialty-specific protocols. For example, it is more common for surgical/orthopedic and neurologic protocols to include mobility as an integral part of treatment or care [23]. In terms of patient characteristics, aspects recognized as highly predictive of in-hospital mobility include pre-admission functional or mobility capability, measured, for instance, as level of dependency according to the Barthel Index Score or indicated by prior use of walking aids [3, 15–19]. However, inconsistencies exist even in evidence of such robust predictors [11, 18]. Similarly, cognitive status on admission has been associated with in-hospital mobility in several studies [11, 16–18], but not in all [19]. In qualitative investigations, having symptoms such as weakness, pain, and fatigue are described by patients as barriers to in-hospital mobility [20, 21]. Patient demographic and clinical characteristics, such as age [16, 18], gender [17, 18], marital status [7], and ethnicity [15], as well as illness severity [18] and physician’s admitting orders, have also been found to play a role in observed mobility [16], but again, not always.

The fact that each study considers a different set of predictors makes both the synthesis of knowledge and the development of personalized treatment and care especially challenging. Therefore, this study examined the degree to which predictors of in-hospital mobility were common across internal medicine units in two different health care systems, Denmark and Israel. We hypothesized that we would find common predictors of in-hospital mobility despite different organizational cultures, different healthcare team characteristics, and different patient personal and cultural features.

## Methods

The study was a post hoc secondary analysis of combined data from two mobility studies, an Israeli prospective cohort [24] and a Danish randomized controlled trial [25].

### Study population

The population in the Israeli study was a subsample from an ongoing prospective cohort study: Hospitalization Process Effects on Mobility Outcomes and Recovery (HoPE-MOR). HoPE-MOR examines diverse risk factors related to the mobility of older adults during acute hospitalization. Eligible patients for the subsample were older adults admitted to one of six general medical inpatient units in two medical centers in Israel [24].

The population in the Danish study consisted of a subsample from a randomized controlled trial: Cross-Continuum Progressive Strength Training in Older Medical Patients–Copenhagen (STAND-Cph). STAND-Cph examined the effects of a strength training program combined with post-training protein supplementation on change in mobility four weeks after discharge in older patients admitted for medical illness. Eligible patients were older adults admitted via the emergency department to one of three medical units in a university hospital in the capital region of Denmark. The details of the study design and methods have been described elsewhere [25].

### Inclusion and exclusion criteria

Both studies recruited older adults (65 +) within the first 24 h of their admission to hospital due to an acute medical condition. The studies applied a similar set of inclusion criteria, inviting cognitively intact patients who were capable of walking with or without walking aids prior to hospitalization and not admitted for end-of-life care to participate. Every eligible patient was invited to participate.

The HoPE-MOR data were collected between February 2018 and May 2019 from all eligible newly admitted older adults. Informed consent was signed by 301 respondents recruited to the research. From the recruited respondents, 95 were excluded due to incomplete data. The final sample included 206 respondents. Excluded participants were similar to participants in demographic and clinical characteristics.

**Fig. 1:**
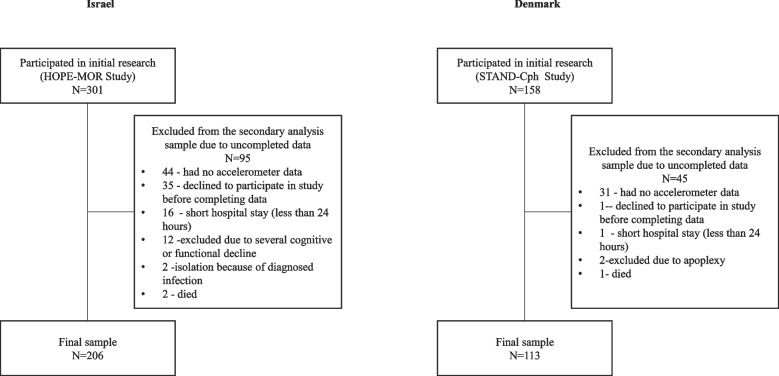
Participant exclusion reasons and numbers from the study in each of study groups

The STAND-Cph data were collected between September 2013 and September 2018 from all eligible newly admitted older adults. One hundred and fifty-eight respondents signed informed consent and were included in this study. From the included respondents, 45 were excluded due to incomplete data. The final sample consisted of 113 respondents. Excluded participants were similar to participants in demographic and clinical characteristics (exclusion process and reasons for exclusion are demonstrated in Fig. 1).

### Measures

#### In-hospital mobility assessment

In-hospital mobility was assessed as number of steps per day determined by activity monitors worn by participants continuously from the time of inclusion in the study until discharge or for seven days (the earlier of the two). In the HoPE-MOR study, in-hospital mobility was assessed by an ActiGraph™ GT9X activity monitor (ActiGraph Corp, LLC, Pensacola, FL) placed on the left ankle. In the STAND-Cph study, in-hospital mobility was measured by an ActivPAL3™ activity monitor (PAL Technologies Ltd., Glasgow, UK) placed on the right mid-thigh.

#### Potential predictors

Our aim was to examine major factors associated with in-hospital mobility. Therefore, we chose variables that assessed similar risk factors in both studies. *Functional status* at time of admission was assessed by the self-reported Independence in Activity Daily Living index (Barthel index ADL) [26] and the de Morton Mobility Index (DEMMI) [27] in both studies. *Cognitive status* was assessed at time of admission using the Mini-Mental State Examination (MMSE) [28] in the HoPE-MOR study and the Orientation Memory Concentration Test (OMC) [29] in the STAND-Cph study. To allow cross-study comparisons, each score was dichotomized to its “cognitive impairment” cut-point [28, 29]. *Community mobility* prior to hospitalization included two indicators: self-reported usage of an assistive walking device (walker) and an item indicating the frequency of going outside dichotomized as going vs not going outside seven times per week or more. The existing evidence supports that use of a walker is associated with restricted ambulation to certain locations, such as the home, and with walking shorter distances [30], and therefor may serve as a proxy for community mobility. *Overall health status*was assessed by a proxy measure: level of pain at admission. We also used parameters retrieved from patients’ medical records – risk of malnutrition (low Body Mass Index) [31], number of comorbidities, and length of hospital stay – as an approximated level of severity of acute illness. *Socio-demographic characteristics* included age, gender, marital status, and community care assistance (paid caregiver provided by social services in the form of at-home assistance with basic and instrumental daily activities).

### Statistical approach

Descriptive statistics for all variables were calculated for each sample separately, and differences were analyzed using a Kruskal Wallis (non-parametric ANOVA) for continuous variables and the Chi-square for categorical variables. Association of sample characteristics with average number of steps was conducted for the two samples using univariate linear regression for continuous variables and point-biserial correlation for categorical variables. We took a conservative approach and used a 0.10 threshold level in the univariate analysis to decide on variable inclusion in the multivariate models. Normality assumption was assessed for continuous variables; this revealed the existence of outliers in number of steps in both samples. In the HoPE-MOR sample, three extreme cases (> = 2.6), and in the STAND-Cph sample, six extremes (> = 2.0) were excluded from the multivariate analysis [32]. Missing values only occurred in the DEMMI in three cases, and these were replaced by imputed data based on multiple implementations on all available variables.

We used multivariate ordinary least squares regression analysis to model predictors of in-hospital mobility assessed by average number of steps separately for the HoPE-MOR and STAND-Cph samples. The multivariate model was based on results of the univariate analysis, with variables entered in the model if they significantly correlated with in-hospital mobility in at least one study sample. All data analyses were performed using IBM SPSS statistical package version 27.0 (IBM, Armonk, NY). In addition, to test comparability of findings, we conducted a sensitivity analysis applying multiple regression to a combined STAND-Cph and HoPE-MOR sample, with an indicator for source of data as an independent dichotomous predictor. For this analysis, we dichotomized variables that were tested using slightly different assessments (cognitive status and community mobility).

## Results

Participants in both study samples were relatively independent in activities of daily living, cognitively intact, and capable of walking independently. Ninety percent walked during their hospital stay. In spite of similar inclusion criteria, the study samples differed in their sociodemographic characteristics (Table 1). Participants in the STAND-Cph study were about two years older, and there was a higher percentage of females. Participants in the HoPE-MOR sample had slightly lower functional status based on subjective and objective evaluations but used fewer walking assistance devices and received less community care. This sample also had a higher average number of comorbidities and suffered more from pain at time of admission. No differences were observed between the samples in mobility level prior to hospitalization, cognitive status, percent with low BMI, or length of stay in hospital.

**Table 1:** Comparison of select sample characteristics for Israel (*N* = 206) and Denmark (*N* = 113)

Variables	HoPE-MOR (Israel)	STAND-Cph (Denmark)	*P* value
Age M ± SD	77.0 ± 7.1	80.7 ± 7.7	< 0.001
Sex: Female N(%)	96(46.6%)	77(68.1%)	< 0.001
Marital status: Married N(%)	109(52.9%)	34(30.1%)	< 0.001
Community care assistance N(%)	66(32.0%)	66(58.4%)	< 0.001
Community mobility: Goes outside 7 times per week or more N(%)	88(42.7%)	55(48.7%)	0.306
Uses walker N(%)	18(8.7%)	34(30.1%)	< 0.001
Cognitive impairment N(%)	35(17.0%)	21(18.6%)	0.720
Number of comorbidities M ± SD	1.7 ± 1.3	1.0 ± 0.8	< 0.001
Independence in ADL (Barthel 0–20) M ± SD	17.4 ± 3.8	18.5 ± 2.2	0.004
Mobility status (DEMMI 0–100) M ± SD	61.1 ± 18.4	66.3 ± 19.6	0.019
BMI < 20 (risk of malnutrition) N (%)	13(6.3%)	9(8.0%)	0.577
Pain on admission (0–5) M ± SD	1.9 ± 1.8	0.7 ± 0.6	< 0.001
Length of stay in hospital M ± SD	6.3 ± 5.9	5.6 ± 4.7	0.283

*ADL* Activity Daily Living index, *DEMMI* de Morton Mobility Index, *BMI* Body Mass Index

The average number of steps was significantly higher in the HoPE-MOR study sample (median (IQR) = 1986.2 (2911.2)) than in the STAND-Cph study sample (median (IQR) = 837.3 (1837.4)). Despite differences in the number of steps, we observed similar correlation patterns (see Table 2). We found significant associations between sample characteristics and a higher number of steps when there was a higher level of community mobility and a higher functional status on admission, while older age, belonging to a lower BMI group (BMI < 20), receiving community care, and using walking devices were associated with fewer steps (see Table 3).

**Table 2:** Association of sample characteristics with average number of steps during hospitalization in Israel (*N* = 206) and Denmark (*N* = 113)

Variables	HoPE-MOR	STAND-Cph
Age	-0.155^**^	-0.283^**^
Sex	-0.108	-0.075
Marital status	0.034	-0.034
Community care assistance	-0.174^*^	-0.207^*^
Community mobility: Goes outside 7 times per week or more	0.200^**^	0.266^**^
Uses walker	-0.232^**^	-0.165^a^
Cognitive impairment	-0.079	-0.171^a^
Number of comorbidities	-0.094	-0.051
Independence in ADL (Barthel 0–20)	0.324^**^	0.305^**^
Mobility status (DEMMI 0–100)	0.341^**^	0.494^**^
BMI < 20 (risk of malnutrition)	-0.201^**^	-0.177^a^
Pain (0–5)	-0.020	-0.136
Length of stay in hospital	-0.231^**^	-0.309^**^

*ADL* Activity Daily Living index, *DEMMI* de Morton Mobility Index, *BMI* Body Mass Index

^a^*p* < 0.1; ^*^p < 0.05; ^**^p < 0.01

**Table 3:** Associations between in-hospital mobility and potential risk factors (combined Israel and Denmark sample *N* = 319)

	1	2	3	4	5	6	7	8	9	10	11
Mean steps	1	-.238^**^	-.233^**^	.196^**^	-.246^**^	-0.106	.266^**^	.334^**^	-.196^**^	0.062	-.227^**^
Age	-.238^**^	1	.302^**^	-.155^**^	.241^**^	.191^**^	-.144^**^	-.259^**^	.160^**^	-0.046	0.074
Community assistance	-.233^**^	.302^**^	1	-.233^**^	.284^**^	.148^**^	-.197^**^	-.233^**^	0.031	0.062	-0.025
Goes to the street 7 times per week or more	.196^**^	-.155^**^	-.233^**^	1	-.159^**^	-0.051	.329^**^	.271^**^	0.066	-.160^**^	-0.092
Uses walker	-.246^**^	.241^**^	.284^**^	-.159^**^	1	0.086	-.330^**^	-.242^**^	0.104	-0.070	0.037
Cognitive impairment	-0.106	.191^**^	.148^**^	-0.051	0.086	1	-.187^**^	-.178^**^	.128^*^	-0.006	.173^**^
ADL (Barthel Index)	.266^**^	-.144^**^	-.197^**^	.329^**^	-.330^**^	-.187^**^	1	.483^**^	-0.073	-.129^*^	-.170^**^
Mobility ability (DEMMI)	.334^**^	-.259^**^	-.233^**^	.271^**^	-.242^**^	-.178^**^	.483^**^	1	0.015	-.179^**^	-.194^**^
Low BMI	-.196^**^	.160^**^	0.031	0.066	0.104	.128^*^	-0.073	0.015	1	-0.086	0.065
Pain	0.062	-0.046	0.062	-.160^**^	-0.070	-0.006	-.129^*^	-.179^**^	-0.086	1	0.103
Length of stay in hospital	-.227^**^	0.074	-0.025	-0.092	0.037	.173^**^	-.170^**^	-.194^**^	0.065	0.103	1

*ADL* Activity Daily Living index, *DEMMI* de Morton Mobility Index, *BMI* Body Mass Index

*p <0.05; **p < 0.01

The multivariate analysis revealed that a higher level of community mobility prior to hospitalization and higher functional status on admission (DEMMI) were significant predictors of a higher number of in-hospital steps, whereas a longer hospital stay was associated with fewer steps in both samples, explaining 28% of the variance in the HoPE-MOR sample and 39% of the variance in the STAND-Cph sample. In HoPE-MOR, having a low BMI was a significant predictor of a lower number of steps. In neither sample was age, community care prior to hospitalization, the use of an assistive walking device, cognitive impairment, or subjective functional status a significant predictor of in-hospital mobility (see Table 4). The sensitivity analysis performed on the combined data showed similar predictors of in-hospital mobility: functional status on admission (DEMMI) (β = 0.258, *p* < 0.001), length of hospital stay (β = -0.165, *p* = 0.001), and low BMI (β = -0.160, *p* = 0.001). Site indicator was also a significant predictor; belonging to the Danish sample was significantly associated with a smaller number of steps (β = -0.276, *p* < 0.001).

**Table 4:** Comparison of the relationship between potential risk factors and in-hospital mobility in Israel (*N* = 206) and Denmark (*N* = 113)

Variables	HoPE-MOR (Israel)		STAND-Cph (Denmark)	
	Standardized Coefficients Beta	*P* value	Standardized Coefficients Beta	*P* value
Age	0.021	0.763	-0.140	0.162
Community care assistance	-0.025	0.725	0.045	0.624
Community mobility (Goes outside 7 times per week or more)	0.215	0.010	0.174	0.041
Uses walker	-0.136	0.093	-0.009	0.928
Cognitive impairment	0.082	0.221	0.010	0.910
Independence in ADL (Barthel 0–20)	-0.014	0.889	0.032	0.735
Mobility status at admission (DEMMI 0–100)	0.215	0.005	0.398	< 0.001
BMI < 20 (risk of malnutrition)	-0.197	0.003	-0.103	0.244
Length of stay in hospital	-0.195	0.003	-0.185	0.040

*ADL* Activity Daily Living index, *DEMMI* de Morton Mobility Index, *BMI* Body Mass Index

## Discussion

In this study, we examined common predictors of in-hospital mobility in two samples of older adults (+ 65) in internal medicine units in Denmark (STAND-Cph) and Israel (HoPE-MOR). We found differences between samples in the number of steps taken during hospitalization. However, in both samples, higher functional status on admission was associated with a higher number of steps, whereas older age, provision of community care assistance, the use of a walker, and a longer hospital stay were associated with fewer steps. Multivariate analyses revealed that a higher level of community mobility prior to hospitalization and higher functional status on admission were common predictors of a higher number of in-hospital steps in both samples. Longer hospital stay (used in this study as a proxy for disease severity) was a significant predictor of a lower average number of in-hospital steps in both samples. Risk of malnutrition was a predictor of a lower number of steps but only in the HoPE-MOR sample.

We found functional status on admission (assessed by the DEMMI) was the strongest predictor of in-hospital mobility across the two samples. Our results are in line with a recent study finding functional mobility assessed by the DEMMI was predictive of higher levels of physical activity during hospitalization [33]. These findings also confirm the results of a previous study in which independence in basic mobility was associated with in-hospital activity [11]. Another recent study [19] of older hospitalized adults found higher levels of functional mobility, assessed by the Short Physical Performance Battery, was predictive of higher levels of physical activity during hospitalization. This is not surprising, as the DEMMI reflects the level of assistance needed to perform basic mobility tasks and thus reflects the ability to independently get out of bed, get in and out of a chair, and walk [34].

A second interesting finding was that the level of community mobility was a predictor of in-hospital mobility. Recent studies [35, 36] of adults aged 65 years and older found the frequency of leaving home and ADL abilities were predictive of one-year hospitalization rate and one-year mortality [35] and lower quality of life [36]. In other words, both studies indicated that community mobility is an important point of interest in a strategy to identify older adults who necessitate attention to avoid functional decline. Our findings further suggests that community mobility can potentially be associated with post-discharge outcomes via its relationship with in-hospital activity.

In previous research, the estimation of actual mobility levels varies, depending on the mobility assessment method used in the study. For example, 12 studies reported in a recent systematic review vary widely in their estimation of the time patients spend in bed, with estimates ranging from 65 to 81% [8]. Further variations appear in reported median number of steps per day: the estimates of different studies of older adults in medical wards range from 656 to 1,791 steps [2, 7, 12, 13]. Variation in step count is at least partially dependent on the type of actigraphy, the duration of monitoring, and the placement of the equipment [14, 37], and the variability makes it difficult to make mobility recommendations and create standardized intervention protocols. Our study sites used different actigraphy devices. According to a review of motion sensors, both sensors we used (ActiGraph and ActivPal) underestimate step counts in frail, older hospitalized patients [38] and tend to undercount steps in slow walkers when worn on the hip (ActiGraph) or thigh (ActivPal). However, in the HoPE-MOR study, the ActiGraph was worn on the ankle, shown to be a more sensitive sensor placement than hip placement [39]. Thus, some of the difference in the number of steps detected in the two samples may be indicative of the sensor placements.

A number of potential predictors of in-hospital mobility were found significant in the univariate, but not the multivariate analyses. Factors that were strong predictors in one analysis may not have been significant in the other. For example, community care assistance, the use of a walker, and baseline ADL function might not be significant in our multivariate analysis because aspects related to dependence in walking are somewhat captured in the DEMMI. This may indicate that when selecting the one tool that will serve as a screening tool, the DEMMI should be considered a leading candidate.

Additional common predictors of mobility and function were not significant in our samples: age, cognitive status, comorbidity, and pain. A possible explanation is that we had relatively homogenous samples with cognitively preserved participants, whose pain level and comorbidity status were relatively low. Pain is often mentioned by patients as a barrier preventing them from being active during hospitalization [20, 21, 40], yet in studies examining multiple factors, this association is not always evident [11, 41]. Nevertheless, these and additional personal and clinical risk factors deserve further consideration in patients with severe health conditions and in frail samples.

### Strengths and limitations

This study featured secondary data analysis using data not originally designed for a comparative investigation. The STAND-Cph sample included participants from a randomized controlled trial, which included an intervention to improve post-hospitalization mobility. However, the primary intervention was conducted between discharge to 4 weeks after hospital discharge, and thus is not expected to have affected the level of in-hospital mobility. This is supported by the fact that we found no between group difference (STAND-Cph intervention versus control) in in-hospital mobility (between group difference: 73 (-482;627), *p* = 0.80) [25]. A potential limitation of this study also is that it did not account for intervening variables during participants' hospital stay that could potentially affect their mobility, including episodes of delirium, tests or procedures requiring rest, or even the dynamic nature of their health condition. This could have affected the ability to detect additional common predictors or dissimilarities. In addition, as different types of scales were used in the Danish and Israeli studies to capture community mobility, we used a single item indicating frequency of going outside to capture this construct. Although this item represents a central aspect in various measures of life space mobility (spatial extent of the person's typical life space) [42], it requires further validation. Nonetheless, the use of common measures and the ability to use comparable samples contribute to the understanding of the universality of the phenomenon of older adults’ in-hospital mobility. Another limitation was the relatively small sample sizes. Moreover, the samples were not representative of all hospitalized older adults (participants were relatively high functioning patients). These factors are likely to affect the generalizability of the findings.

## Conclusion

Our study investigated predictors of in-hospital mobility in two different health care systems. We had a unique opportunity to compare a phenomenon in similar populations and settings using similar predictors. This cross-country study adds to the literature by reinforcing the universality of the in-hospital mobility phenomenon and showing that pre-hospitalization and baseline mobility functions are the strongest predictors of in-hospital mobility in different care environments and populations. The study highlights the robustness of the DEMMI as a predictor of in-hospital mobility; our findings suggest it should be included in older adults' clinical assessment to identify at-risk patients early during their hospital stay. The study provides new insights into common predictors of in-hospital activity, raising important points to consider when older adults are admitted to the hospital for acute care.

## Data Availability

The datasets used during the current study available from the corresponding author on reasonable request.

## Abbreviations

HoPE-MOR: Hospitalization process effects on mobility outcomes and recovery
STAND-Cph: Strength training in older medical patients–Copenhagen
MMSE: Mini-mental state examination
OMC: Orientation memory concentration test
ADL: Activity daily living index
DEMMI: De Morton mobility index
BMI: Body mass index
IQR: Interquartile range
